# School-Based Stroke Education Through On-Demand E-learning During Coronavirus Disease 2019 Pandemic: Itoigawa Stroke Awareness Campaign

**DOI:** 10.7759/cureus.37380

**Published:** 2023-04-10

**Authors:** Masahito Katsuki, Junko Kawahara, Hiroyuki Senda, Chinami Yamagishi, Satoshi Mizusawa, Yasuhide Ueki, Shin Kawamura, Kenta Kashiwagi, Akihito Koh, Rie Hashiba, Atsuko Ono, Yuki Watabe, Kazuhiro Ando, Bumpei Kikuchi, Shinya Yamashita, Fuminori Yamagishi

**Affiliations:** 1 Department of Neurosurgery, Itoigawa General Hospital, Itoigawa, JPN; 2 Department of Health Promotion, Itoigawa City Servant Service, Itoigawa, JPN; 3 Department of Fire, Itoigawa City Servant Service, Itoigawa, JPN; 4 Board of Education, Itoigawa City Servant Service, Itoigawa, JPN; 5 Department of Neurology, Itoigawa General Hospital, Itoigawa, JPN; 6 Department of Neurosurgery, Niigata Prefectural Central Hospital, Joetsu, JPN; 7 Department of Surgery, Itoigawa General Hospital, Itoigawa, JPN

**Keywords:** effects of social media, digital transformation (dx), efficient medicine, awareness, stroke, coronavirus disease 2019 (covid-19), artificial intelligence

## Abstract

Introduction

Raising stroke awareness is important to shorten the interval from onset to consultation. We performed a school-based stroke education by on-demand e-learning during the coronavirus disease 2019 pandemic.

Methods

We performed on-demand e-learning and distributed the online- and paper-based manga about stroke for students and parental guardians in August 2021. We carried out this in a manner similar to the prior effective online stroke awareness initiatives in Japan. An online post-educational survey in October 2021 was conducted to evaluate the awareness effects by asking participants about their knowledge. We also investigated the modified Rankin Scale (mRS) at the discharge of stroke patients who were treated in our hospital during the before- and after-campaign periods, respectively.

Results

We distributed the paper-based manga and asked to work on this campaign to all 2,429 students (1,545 elementary school and 884 junior high school students) who lived in Itoigawa. We acquired 261 (10.7%) online responses from the students and 211 (8.7%) responses from their parental guardians. The number of students who chose all correct answers in the survey significantly increased after the campaign (205/261, 78.5%) compared to that before the campaign (135/261, 51.7%) and those of parental guardians showed similar trends (before campaign 93/211, 44.1%; after campaign 198/211, 93.8%). We investigated 282 stroke patients (90 patients before and 192 patients after-campaign period), and their mRS at discharge after-campaign seemed to be improved.

Conclusion

Only 10.7% of students and 8.7% of the parental guardians worked on the online survey. However, the number of those who chose correct answers about stroke increased after the campaign. After this campaign, the mRS of stroke patients at discharge improved although it was unclear if this is a direct result of this activity.

## Introduction

To improve stroke outcomes, it is crucial to shorten the interval between the onset of the stroke and hospital admission. A multilayer teaching program for the general public, emergency medical technicians, and hospital staff improved the accuracy of stroke diagnosis, which led to a rise in the number of patients presenting for examination inside the therapeutic window for acute thrombolytic therapy [1]. Also, stroke education for school children and students has been demonstrated to be effective for both the students and their families through communication among family members [2-7]. In other words, when disease education is provided to school students, the effects have been shown to ripple out to their families. However, it is difficult under the coronavirus-2019 (COVID-19) pandemic for medical professionals to educate students about stroke in school in person. This is because schools have sometimes been closed, and face-to-face contact has been restricted due to the COVID-19 pandemic. Beginning in 2021, one tablet terminal was loaned to each student, and interactive remote learning was conducted in Japan [8]. Using this network system, we have succeeded in a headache and migraine awareness campaign [9]. Similar to this, we performed a school-based stroke education by on-demand e-learning during the COVID-19 pandemic, entitled "Itoigawa Stroke Awareness Campaign." We herein report the awareness effects and the outcomes of stroke treatment at our hospital, the only stroke hospital in this area.

## Materials and methods

Campaign procedure

We performed this stroke awareness campaign prospectively from January to December 2021. This campaign was held in Itoigawa city, Niigata, Japan, by Itoigawa General Hospital. Itoigawa city is one of the rural cities in Japan. In January 2021, the overall population of Itoigawa city was 40,375. The working-age population was 20,458 (50.1%), and the aged population was 16,330 (40.0%). The population density is 54.1 people/km^2^, much smaller than those of Tokyo (6,168.2 people/km^2^) and Osaka (4,640.0 people/km^2^). Itoigawa city is experiencing a significant population decline, especially among the 15-39 age group, and is facing problems maintaining its future industry. Primary and secondary industries account for 5.1% and 35.5% of the city's population, respectively. Itoigawa General Hospital is the only hospital that admits and treats strokes in patients who were independent before the stroke onset. Patients who are already admitted to geriatric hospitals or require assistance cannot be brought to our hospital when they have a stroke.

We first educated non-stroke-specialized doctors, hospital staff, and emergency medical technicians through on-demand e-learning in January 2021. All staff involved in stroke care completed the viewing. The contents were based on the Immediate Stroke Life Support Course [10]. Then, we performed on-demand e-learning for students and parental guardians in Itoigawa in August 2021. We used the same videos, cartoons, and leaflets to make awareness, and questionnaire items to evaluate the awareness performance that the previous stroke awareness campaign in Japan [2-7] used. After learning about the tools and lecturing method from CY, one of the instructors of the previous stroke awareness campaign in Japan [2-7], we performed this campaign. The only difference is that these were conducted on-demand and online rather than in person.

Participating students received a 40-min online lesson about stroke made by Google Forms. Sheets of papers explaining this initiative and a two-dimensional code to access Google Forms were distributed to all students and parents. It was not a direct lecture but a system of lesson videos and questionnaires as participants proceeded through Google Forms. We asked schoolteachers to inform the students and parents about this. This campaign was an extracurricular school activity, and families and children could work on it at home at any time they wanted (on demand). They were informed that they could work on it at their own discretion.

First, we performed a pre-educational survey through Google Forms for the students and the one parental guardians (10 min). The items we asked for are shown in Table 1.

**Table 1:** Items in the questionnaire sheet and percentages of correct responses

	Students n=261					Parental guardian n=211				
Stroke signs and symptoms (three of seven items were correct)	Before campaign		After campaign			Before campaign		After campaign		
Correctly choosing correct answers	n	%	n	%	p value	n	%	n	%	p value
facial weakness in one side	219	83.9%	251	96.2%	<0.001	185	87.7%	207	98.1%	<0.001
speech disturbance	242	92.7%	243	93.1%	0.701	191	90.5%	189	89.6%	0.798
numbness in one side of the body	227	87.0%	232	88.9%	0.566	186	88.2%	191	90.5%	0.445
Correctly choosing incorrect answer										
Flatus	11	4.2%	12	4.6%	0.887	12	5.7%	3	1.4%	0.034
Fever	39	14.9%	18	6.9%	0.013	19	9.0%	15	7.1%	0.499
stomachache	13	5.0%	7	2.7%	0.201	6	2.8%	2	0.9%	0.169
stiff shoulders	40	15.3%	12	4.6%	<0.001	37	17.5%	15	7.1%	0.004
Risk factors for stroke (four of six items were correct)										
Correctly choosing correct answers										
hypertension	205	78.5%	215	82.4%	0.301	198	93.8%	201	95.3%	0.580
high cholesterol	198	75.9%	229	87.7%	<0.001	185	87.7%	180	85.3%	0.499
smoking	206	78.9%	231	88.5%	0.017	186	88.2%	189	89.6%	0.678
heavy drinking	224	85.8%	239	91.6%	0.062	178	84.4%	196	92.9%	0.012
Correctly choosing incorrect answer										
back pain	10	3.8%	12	4.6%	0.702	12	5.7%	9	4.3%	0.569
constipation	21	8.0%	16	6.1%	0.412	16	7.6%	9	4.3%	0.198
Attitude towards stroke (one of four items was correct)										
Call an ambulance	228	87.4%	240	92.0%	0.104	199	94.3%	201	95.3%	0.133
Take them to visit the hospital by taxi or private car/ Take them to visit family doctor/ Lie down to rest										
Correctly choosing all correct and incorrect answers	135	51.7%	205	78.5%	<0.001	93	44.1%	198	93.8%	<0.001

**Figure 1:**
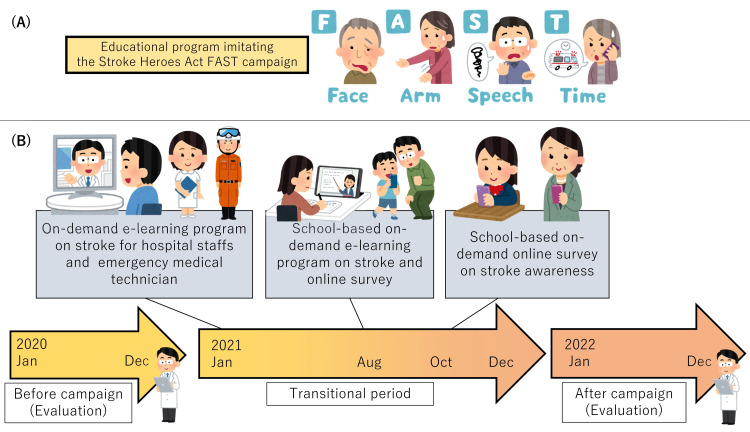
Study design (A) FAST mnemonic. Facial numbness, Arm or leg numbness or weakness, difficulty in Speaking or understanding, and Time to call an ambulance. (B) Timeline of the campaign. The students and their parents were asked to work on a pre-educational survey and e-learning on-demand and online in August 2021. We performed an online post-educational survey in October 2021. The primary outcomes were the percentage of correct questionnaire answers. The secondary outcomes were the percentages of three-month favorable outcomes of the stroke patients who were treated in our hospital. We investigated them during the before-campaign period (January to December 2020) and after-campaign period (January to December 2022), respectively.

Then, the students read manga (comic books) [4], watched animation videos [7], and video lessons from a neurosurgeon [6]. Students were initially instructed about risk factors and signs of stroke through video lessons by a neurosurgeon (15 min). Finally, they watched an animated cartoon and reviewed the stroke sign using the FAST mnemonic [11,12] (Facial numbness, Arm or leg numbness or weakness, difficulty in Speaking or understanding, and Time to call an ambulance, 15 min) using manga (Figure 1A).

The students and their parents were asked to watch the movie together any time they wanted on-demand in August 2021. Furthermore, in parallel with online education, we distributed the paper-based manga to all the students and parental guardians in Itoigawa city. For those who are too busy to work on this campaign, we urged them to read online- or paper-based manga or online animation video alone.

We performed an online post-educational survey in October 2021; We again asked the students and the one parental guardian to fill out the same online survey (Table 1) as a result of the stroke awareness campaign. Valid responses were those that filled in all the items on the survey sheet and who answered in both the pre- and post-educational surveys. Therefore, there were no missing data in the valid responses. We confirmed not to sample the same person more than once by making the survey able to be answered only once through Google Forms. Unlike the headache awareness we performed before [9], we did not repeatedly call for participation in this stroke awareness campaign.

Measuring effectiveness

The primary outcomes were the percentage of correct questionnaire answers. It contains the topic of stroke signs, symptoms, and risk factors as multiple choice, attitude toward stroke, and behavior regarding risk factors (Table 1). Participants were assessed whether they correctly checked each item and selected all correct answers (full points). The pre-educational ratio and post-educational ratio of the correct answers were compared.

The secondary outcomes were the modified Rankin Scale (mRS) at three months of the stroke patients who were treated in our hospital. The investigated patients were stroke patients, including ruptured cerebral aneurysm, intracranial hemorrhage due to hypertension and other vascular diseases, and cerebral infarction. Trauma, functional diseases, transient ischemic attack, and cerebral tumors were excluded. All patients had been mRS 0 to 2 before admission. We investigated them during the before-campaign period (12 months; January to December 2020) and after-campaign period (12 months; January to December 2022), respectively, and compared them. The treatment strategy was decided based on the Japanese Guideline for the Management of Stroke 2021 [13]. In our hospital, we can perform medication, neurosurgical treatment with craniotomy, endoscopy [14], and recombinant tissue-type plasminogen activator (rt-PA) infusion therapy. When endovascular therapy is required, we transport the patients to the nearest stroke center where it can be performed. The time course of this campaign is shown in Figure 1B.

Statistical analysis

The actual number and frequency (%) were shown. Variables without a normal distribution are shown as the median (interquartile range). Chi-square, McNemar's and Mann-Whitney U tests were used appropriately. Statistical significance was defined as one-tailed p < 0.05. We used SPSS 28.0.0 (IBM Corp., Armonk, NY, USA) for statistical analysis.

Ethical aspects

The Itoigawa General Hospital Ethics Committee approved this study (approval number: 2021-4, 2021-5). The answers collected on the online survey were anonymous. We never forced the respondents to participate in this awareness campaign or survey; we only invited them to participate of their own free will. It was explicitly stated in the online survey that by answering the questions, they were giving their consent to this study. The purpose of the study was also explained to the participants in writing and handed to them. If they were unable or did not want to participate, they were asked to submit a blank sheet, thus providing an opportunity for non-participation. The requirement for written informed consent from stroke patients was waived because of the study’s retrospective nature. Instead, opt-out consent documents were presented on the Itoigawa General Hospital website (https://www.itoigawa-hp.jp/other/rinri/img/20220926_optout.pdf) for patients who did not wish to participate. All methods were performed following the relevant guidelines and regulations of the Declaration of Helsinki.

## Results

We distributed the paper-based manga and asked all 2,429 students (1,545 elementary school and 884 junior high school students) who lived in Itoigawa to work on this campaign. We acquired 261 (10.7%) online responses from the students and 211 (8.7%) responses from their parental guardians. We could not know how many students and their parental guardians only read manga or watched animation videos without working on the online survey. Regarding the primary outcome, the number of students who chose all correct answers (full points) significantly increased after the campaign (205/261, 78.5%) compared to that before the campaign (135/261, 51.7%) (p<0.001) and those of parental guardians showed similar trends (before campaign 93/211, 44.1%; after campaign 198/211, 93.8%) (p<0.001). The detail is described in Table 1.

As the secondary outcome, mRS at discharge was investigated. The results of 282 patients (90 patients before and 192 patients after-campaign period) were described in Table 2.

**Table 2:** Patients characteristics and ordinal logistic regression results JCS; Japan Coma Scale, ｍRS; modified Rankin Scale

Variables	Before-campaign (n=90)	After-campaign (n=192)	Total
Women: Men (%women)	27:63 (30.0%)	71:121 (34.8%)	198:98 (65.2%)
Age	75 (15)	77 (15)	76 (15)
Prestroke mRS	0 (2)	0 (2)	0 (2)
mRS 0	46 (51.1%)	126 (65.6%)	172 (61.0%)
mRS 1	19 (21.1%)	10 (5.2%)	29 (10.3%)
mRS 2	25 (27.8%)	56 (29.2%)	81 (28.8%)
JCS on admission			
JCS 0	27 (30.0%)	76 (39.6%)	103 (36.5%)
JCS 1-3	43 (47.8%)	86 (44.8%)	129 (45.7%)
JCS 10-30	8 (8.9%)	20 (10.4%)	28 (9.9%)
JCS 100-300	12 (13.3%)	10 (5.2%)	22 (7.8%)
Before or After the campaign	90 (31.9%)	192 (68.1%)	282 (100.0%)
Stoke type			
Subarachnoid hemorrhage	11 (12.2%)	8 (4.2%)	19 (6.7%)
Intracranial hemorrhage	26 (28.9%)	33 (17.2%)	59 (20.9%)
Cerebral infarction	53 (58.9%)	151 (78.6%)	204 (72.3%)
mRS at discharge	3 (3)	2 (3)	3 (3)
mRS 0	3 (3.3%)	31 (16.1%)	34 (12.1%)
mRS 1	13 (14.4%)	24 (12.5%)	37 (13.1%)
mRS 2	20 (22.2%)	49 (25.5%)	69 (24.5%)
mRS 3	19 (21.1%)	26 (13.5%)	45 (16.0%)
mRS 4	12 (13.3%)	29 (15.1%)	41 (14.5%)
mRS 5	9 (10.0%)	14 (7.3%)	23 (8.2%)
mRS 6	14 (15.6%)	19 (9.9%)	33 (11.7%)

**Figure 2:**
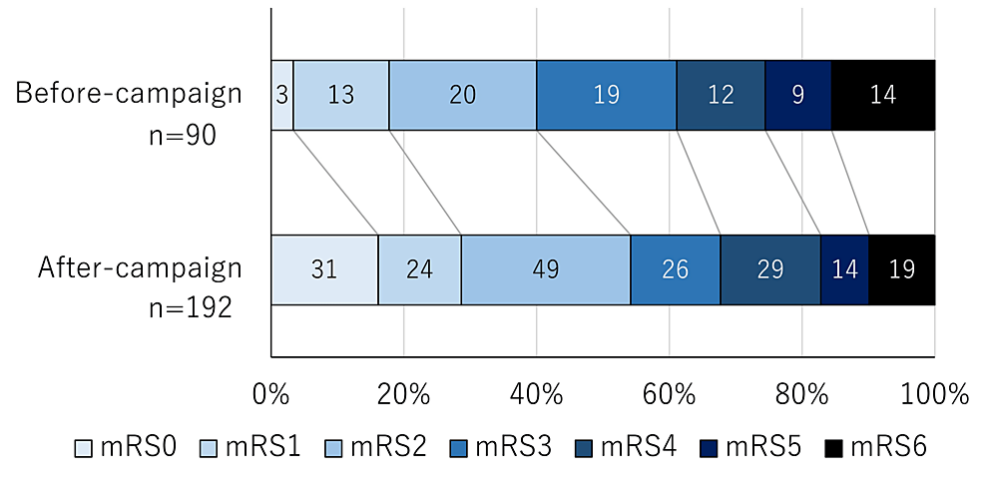
Modified Rankin Scale at discharge A modified Rankin Scale (mRS) score at discharge is shown. mRS during the after-campaign period was significantly better than that during the before-campaign period (p=0.013).

The median age was 76 (15), and 198 (65.2%) were women. The median pre-stroke mRS was 0 (2). Patients with JCS 0 on admission were 102 (36.5%), those with JCS 1-3 were 129 (45.7%), those with JCS10-30 were 28 (9.9%), and those with JCS 100-300 were 22 (7.8%). Subarachnoid hemorrhages were 19 (6.7%), intracranial hemorrhages were 59 (20.9%), and cerebral infarctions were 204 (72.3%). The median mRS at discharge was 3 (3), and detail is described in Figure 2.

mRS at discharge after-campaign was significantly better than before (p=0.013). Recombinant tissue-type plasminogen activator treatment was performed more frequently after this campaign (35/192, 18.2%) than before the campaign (5/90, 5.6%) (p=0.003), and the door-to-needle time improved from 148 (60-182) minutes to 40 (18-128) minutes (p=0.016). The number of percutaneous mechanical thrombus retrieval procedures also increased after the awareness campaign from 2/90 (2.2%) to 10/192 (5.2%), but not statistically significant (p=0.349).

## Discussion

We prospectively performed the stroke awareness campaign and distributed online and paper-based manga and on-demand e-learning content to all the students and their parental guardians. Unfortunately, only 10.7% of students and 8.7% of the parental guardians worked on the online survey. However, among those, the number of those who chose correct answers about stroke increased after the campaign.

In general, the quality of medical care is lower in rural areas than in urban areas. The areal deprivation indexes (ADIs) indicate the level of socioeconomic status of districts. Higher ADIs are associated with higher stroke risk [15] and worse outcomes [16]. The hazard ratio of stroke incidence is 1.12-1.19 in the areas with higher ADIs in Japan [15]. Not only is accessibility to advanced medical care low in the area with higher ADI. In such areas, it is inferred that irrational behavior is the reason for worse stroke risk and outcomes. People do not call an ambulance when they have a stroke because they are concerned about their surroundings, or because they decide that they will recover if they just stay at home and watch their condition [17].

Fukuda [17] investigated 3,902 patients with stroke who were administered and treated in Kochi prefecture, Japan. Patients who took more than four hours from the onset of illness to the first visit to the hospital (admission to the first care hospitals) were defined as “delayed visit,” and the association between ADI and a delayed visit was examined in the municipality where the corresponding patients resided in. The ADI was calculated from the ratio of elderly couple households, elderly single-person households, single-mother households, rental housing, service industry workers, agricultural workers, blue-collar workers, and the unemployment rate in the region [18]. ADI indicates the difficulty of obtaining good quality “connections” between the individual and the people around them, the community, services, etc. The higher ADI, the more disadvantaged the individual is judged to be socioeconomic. ADI is not directly related to income or assets but indicates whether the connections are good quality. The study showed a significant association between the delay in receiving medical examinations and ADI: a high ADI led to a delay in receiving medical examinations.

The mean (standard deviation, range) ADI in 2010 Japan was 6.19% (0.74%, 6.3-18.0%) and that of Itoigawa was just under 10% [19], suggesting higher ADI. In areas with high ADI, relationships are generally considered narrow and dense, and a diversity of information is likely to be lacking. As a result, they may not have the opportunity to correct their erroneous values about emergencies, such as being embarrassed if others know about them, or their adherence to the bias that they will feel better after a night's sleep, which may prevent them from taking appropriate action in an emergency. Therefore, a stroke awareness campaign is needed to have them know the correct information in high ADI areas.

Although this is only speculation, the increase in the number of stroke patients who visited our hospital after the awareness campaign and the increase in the percentage of patients who received rt-PA suggest that the ratio of reasonable medical consultation behavior and the speed at which patients were seen increased. In the future, it is necessary to examine the actual time from detection to consultation and the psychological factors that caused patients to visit the hospital to consider the awareness effects.

Stroke awareness activities have been around for a long time [1], and the materials and other resources have been enhanced. The problem is how to distribute this correct information to the target population [2-7]. To educate the elderly, who are the most vulnerable age group to stroke, it may be possible to distribute materials and give lectures at meetings where the elderly gather. However, this would be very costly, and it would be nearly impossible to reach every community. Therefore, the information was disseminated online by the school. Children and their parental guardians, who seem to find a stroke victim and call the emergency, could have got the information this way. However, this awareness campaign has not reached households where only the elderly live. In the future, it will be necessary to consider ways to deliver the information more widely in cooperation with the government, for example, by mail or at meetings, combined with online education.

We mention future prospects. Under the COVID-19 pandemic, our stroke awareness campaign can be performed without face-to-face communication to avoid infection. Also, our materials used for awareness raising can be used online without paper-based materials. They can be installed into smartphone applications and further spread on social media, leading to strong influence not only in our area but also worldwide. Also, our method of raising awareness is online and does not directly utilize human materials. It could be used to further raise awareness in areas where telestroke is being done, such as telemedicine [20] and remote island medicine [21]. Correcting regional disparities in stroke care by healthcare providers and medical resources is of course important, but updating residents' knowledge will also need to be done in parallel.

This study has some limitations. First, our study can have responder bias due to the collection method employed. Therefore, the results did not necessarily reflect those of the general population of students and their parental guardians. Second, the collection rates were only about 10%. It was suggested that a certain number of people who were not interested in stroke did not participate in the on-demand survey, even if it was a school initiative, the same as the previous campaign [9]. Third, no direct study has been conducted to determine whether awareness-raising has reduced the actual time from stroke onset to arrival at the hospital. Fourth, the number of stroke patients has nearly doubled after the awareness campaign. This is not only because of the increase in the number of patients seen as a result of the awareness campaign. There are regional reasons for this; stroke patients with mild symptoms had received outpatient care at a nearby doctor if they did not call for emergency care and ambulance or had been seen by a department other than the stroke (neurosurgical) department. It is undeniable that the improvement in mRS at discharge from the hospital is because stroke specialists now see many patients due to the awareness campaign. We should investigate the detail in the next campaign; increased transport speed by emergency services, faster detection of strokes and increased consultation rates, the increased willingness to perform rt-PA therapy, or education for school children. Fifth, a smaller number (about 10%) participated in the online survey, but the actual number of those who viewed online content, such as animation videos and manga, is unknown. Whether paper-based manga or online content contributed more to raising awareness is not certain. Another limitation is that generally, the education interventions do not last long in terms of results. Therefore, it would be a good idea to re-evaluate knowledge retention at a later time, and we will perform it in the next stroke awareness campaign.

## Conclusions

We prospectively performed Itoigawa Stroke Awareness Campaign and distributed paper-based manga and on-demand e-learning content to all the students and their parental guardians. Unfortunately, only 10.7% of students and 8.7% of the parental guardians worked on the online survey. However, among those, the number of those who chose correct answers about stroke increased after the campaign. Furthermore, this campaign improved three-month mRS at our hospital of stroke patients although it was unclear if this is a direct result of this activity. Under the COVID-19 pandemic, it is difficult for medical professionals to educate students about stroke in school in person. However, on-demand e-learning supported by distributing online- and paper-based manga on stroke may potentially raise awareness of stroke.
